# A Computational Study on Halogen/Halide Redox Mediators and Their Role in ^1^O_2_ Release in Aprotic Li–O_2_ Batteries

**DOI:** 10.1021/acs.jpca.3c05246

**Published:** 2023-10-27

**Authors:** Adriano Pierini, Angelica Petrongari, Vanessa Piacentini, Sergio Brutti, Enrico Bodo

**Affiliations:** † Chemistry Department, University of Rome “La Sapienza”, P. A. Moro 5, 00185 Rome, Italy

## Abstract

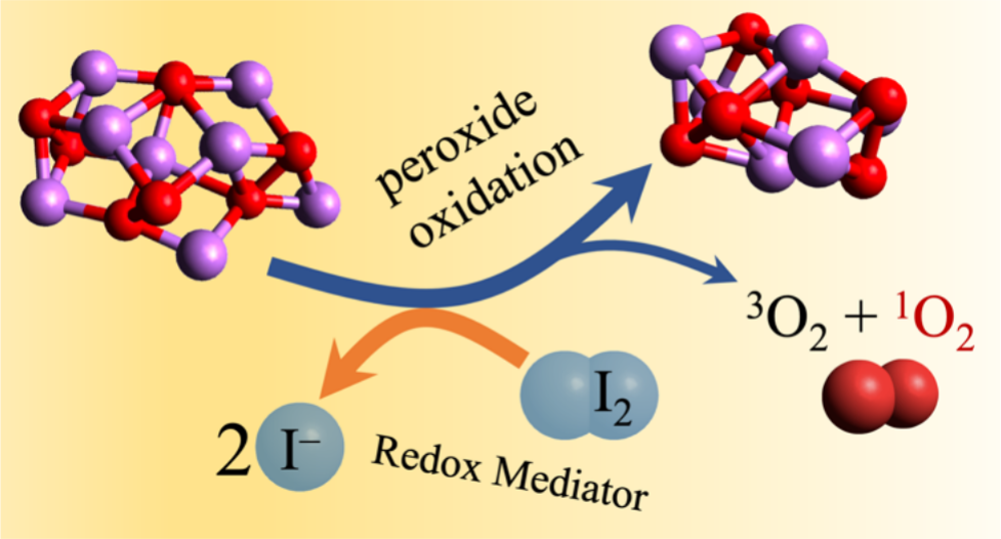

We present a computational study on the redox reactions of small clusters of Li superoxide and peroxide in the presence of halogen/halide redox mediators. The study is based on DFT calculations with a double hybrid functional and an implicit solvent model. It shows that iodine is less effective than bromine in the oxidation of Li_2_O_2_ to oxygen. On the basis of our thermodynamic data, in solvents with a low dielectric constant, iodine does not spontaneously promote either the oxidation of Li_2_O_2_ or the release of singlet oxygen, while bromine could spontaneously trigger both events. When a solvent with a large dielectric constant is used, both halogens appear to be able, at least on the basis of thermodynamics, to react spontaneously with the oxides, and the ensuing reaction sequence turned out to be strongly exoergic, thereby providing a route for the release of significant amounts of singlet oxygen. The role of spin–orbit coupling in providing a mechanism for singlet–triplet intersystem crossing has also been assessed.

## 1 Introduction

Aprotic lithium–oxygen batteries (LOBs), based on the (electro)reduction of molecular oxygen at a porous cathode, are a key topic in the search for secondary batteries with higher energy density.^1−6^ However, in order to have practical LOBs with acceptable stability and cycle life, major challenges are still to be solved.^1,2,6−9^ Parasitic reactions are well-known to undermine the long-term stability of LOBs, leading to progressive degradation of the electrolyte and cell failure.

The release of molecular oxygen in its first excited electronic state, commonly known as “singlet oxygen” (^1^O_2_), is now broadly recognized to be a major source of uncontrolled side reactions in LOBs.^8,10−13^ Large charge overpotentials, mostly due to the electrically insulating nature of the peroxide discharge products, also heavily impact the reversibility of the battery. These two problems, namely, the parasitic reactivity and the overpotentials, are mutually related and self-nourishing. In fact, the deposition on the electrode surface of side-reaction products triggered by ^1^O_2_ worsens the electrical conductivity at the electrode–electrolyte interface, while at the same time, the application of high voltages push the cell materials up to their electrochemical stability limit, thus reaching the onset for more degradative processes.^14^

The addition of soluble redox catalysts, usually called “redox mediators” (RMs), into the electrolyte is a promising strategy to mitigate the impact of high overpotentials.^9,15,16^ They act by chemically oxidizing the discharge product, while the resulting reduced form of the RM undergoes electrochemical oxidation at the electrode to restore the oxidized RM and repeat the cycle. The net result is that the battery can ideally be charged at a working voltage equal to the redox potential of the RM^(ox)^/RM^(red)^ couple, which is typically slightly above the thermodynamic oxidation potential of Li_2_O_2_ to give O_2_ (oxygen evolution reaction, OER)

Many different classes of organic, inorganic, and organometallic chemical compounds^17^ have been studied as suitable RMs for LOBs. Among them, the use of redox couples based on the different oxidation states of iodine/iodide species has been thoroughly reported and, to a much lesser extent, also those based on bromine/bromide species.

On the other hand, RMs can affect the impact of singlet oxygen inside the cells through different mechanisms. In the first place, the most compelling reason for using redox catalysts for recharging the battery is precisely the reduction of the overpotentials for reversibly oxidizing the discharge products. In fact, the application of large voltages provides the chemical energy required for releasing ^1^O_2_. A charging voltage of about 3.5–3.6 V vs Li represents a threshold for the release of significant amounts of singlet oxygen, which is formed upon direct, nonmediated oxidation of Li_2_O_2_ on carbon-based electrodes.^10,18−20^ Moreover, RMs can interact with already formed ^1^O_2_ either by chemical reaction, which progressively destroys the catalytic amount of the mediator, or by physical quenching. In the latter case, singlet oxygen gets deactivated to the triplet state without affecting the chemical nature of the RM. This usually happens, as with other quenchers, by the formation of an intermediate charge-transfer complex which can favorably decay to the ground-state multiplicity via a radiationless spin transition.^21^

The multiple roles of RMs interplay with the additional complexity of the OER. In fact, the electrochemical OER in LOBs follows a complex mechanism during charge. Instead of a direct two-electron oxidation of Li_2_O_2_ to give O_2_,^22,23^ experimental evidence demonstrates a multistep mechanism.^24−26^ In the first step, the discharge product undergoes a progressive delithiation, which leads to a mixed superoxide/peroxide phase (Li_2*n*–*x*_O_2*n*_ or, alternatively, (LiO_2_)_*x*_(Li_2_O_2_)_*n*−*x*_):
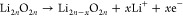
1Subsequently, the newly formed superoxides can be oxidized again (eq 2), or they can spontaneously disproportionate (eq 3):
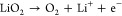
2
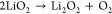
3Similar sequences of one-electron reactions have also been observed for RM-assisted peroxide oxidative decomposition.^11^

Iodine and bromine are added to the electrolyte in their reduced forms (halides), and both can be oxidized at the electrode (typically to I_2_ and Br_2_) at redox potentials slightly above 3.0 V vs Li and then partake in the oxidation of the peroxides in LOBs. Here we focus on the analysis of the redox mediation mechanism of iodine and bromine in the oxidation of lithium peroxide. In particular, we use theoretical calculations to investigate different reactive pathways of iodine- and bromine-based mediation of the OER. Previous (electro-)kinetic studies highlight that with most RM classes, the electron-transfer steps take place as inner-sphere processes.^27^ In analogy with redox reactions of transition-metal complexes, this reactive step typically involves the transfer of a bridging unit (e.g., a ligand, in the case of coordination complexes) between the two redox-active centers. Therefore, we limited our focus to those oxidation pathways where the electron transfer from the discharge products to an oxidized form of the RM is accompanied by Li^+^ abstraction, which preserves the electroneutrality of the species:
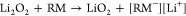
4
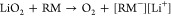
5where RM and RM^–^ species in eqs 4 and 5 generically stand for the oxidized and reduced forms of the redox mediator, respectively.

## 2 Methods and Model System Composition

Standard reaction free energies (Δ_r_*G*°) were calculated ab initio for different combinations of RMs and stoichiometries of the discharge products. These calculations were performed using the B2PLYP functional^28−30^ with the all-electron DKH-def2-TZVPP basis for elements up to Br and the SARC-DKH-def2-TZVPP basis for I.^31^ The functional choice was supported by comparison with an ab initio CCSD(T) reference calculation (see Supporting Information (SI) section S3). The DKH2 relativistic Hamiltonian was applied to account for relativistic scalar corrections to the energy. We used the ORCA package distribution (version 5.03^32^) for all calculations.

For the halogen/halide RMs, many redox couples were considered, based on different oxidation states of the halogen species X = Br, I. Those that are relevant for the discussion are
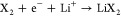
6
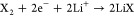
7
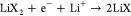
8
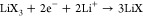
9Considering the low solubility of peroxides in typical organic solvents, a cluster model was adopted for the discharge products (LiO_2_)_*x*_(Li_2_O_2_)_*n*−*x*_ while avoiding the computational overhead due to a periodic solid-state simulation of large slabs with absorbed molecules. This approach has already been used with profit in previous computational studies on lithium peroxide oxidation,^33,34^ and it was shown that small clusters made up of four Li_2_O_2_ units already provide a decent approximation to the electronic structure of larger, nanosized molecular clusters.^35^

In order to produce reasonable geometries for each cluster stoichiometry, a set of initial random configurations (ca. 10–12) were preliminarily optimized at the semiempirical GFN2-xTB method.^36^ Among these minimum structures, those lying within 0.05*E*_h_ from the lowest one were reoptimized by DFT as described above. The lowest-energy structure was finally selected for the free energy evaluation by using a standard Hessian calculation. All calculations were initially performed in the gas phase; then both optimization and frequency calculations were repeated with implicit solvents. For the solvent, we opted for two SMD models,^37^ one with the parameters for DMSO and the other with those for diethyl ether. The latter was chosen to mimic the glyme ethers (such as DME and TEGDME) commonly employed in Li–O_2_ batteries. The geometries optimized in implicit solvents do not present important differences compared to the gas-phase ones. These last are reported in SI section S2.

Spin–orbit coupling (SOC) calculations were performed on X_2_–Li_2_O_2_ systems at the TDDFT level in order to evaluate the mixing between triplet and singlet spin states along normal vibrational modes that are strongly coupled with the oxygen-to-halogen electron transfer. More details on these calculations are reported in SI section S1.

## 3 Results and Discussion

### 3.1 Elementary Reactions

In line with the general consensus, we modeled the oxidation of peroxides through a sequence of one-electron transfers. Under this hypothesis, the following peroxide/superoxide clusters were selected as reactants, intermediates, and products along the oxidation pathways:

- **P4**: four peroxide units: (Li_2_O_2_)_4_
- **SP3**: one superoxide and three peroxide units: (LiO_2_)(Li_2_O_2_)_3_
- **S2P2**: two superoxide and two peroxide units: (LiO_2_)_2_(Li_2_O_2_)_2_
- **P3**: three peroxide units: (Li_2_O_2_)_3_

These clusters and the redox couples of eqs 6–9 were combined into a set of reactions in the form of eqs 4 and 5, and the reaction Gibbs free energies were computed. The results are reported in Table 1 for iodine and in Table 2 for bromine.

**Table 1:** Iodine RM Oxidation Reactions: Computed Δ_r_*G°* (B2PLYP Triple-ζ Basis Set, in eV) in Different Solvents

no.	reaction	Δ_r_*G*° (gas-phase)	Δ_r_*G*° (ether)	Δ_r_*G*° (DMSO)
Peroxide Oxidation (1e^–^)				
*i1*	I_2_ + **P4** → LiI_2_ + **SP3**	+0.54	+0.30	+0.16
*i2*	I_2_ + **SP3** → LiI_2_ + **S2P2**	+1.08	+0.91	+0.80
*i3*	I_2_ + **P4** → 2LiI + **S2P2**	+1.71	+0.59	+0.14
*i4*	LiI_2_ + **P4** → 2LiI + **SP3**	+0.64	–0.32	–0.66
*i5*	LiI_2_ + **SP3** → 2LiI + **S2P2**	+1.17	+0.29	–0.02
*i6*	LiI_3_ + **P4** → 3LiI + **S2P2**	+ 1.62	+0.40	–0.02
Superoxide Oxidation (1e^**–**^**)**				
*i7*	I_2_ + **SP3** → LiI_2_ + **P3** + O_2_	+1.39	+0.90	+0.64
*i8*	LiI_2_ + **SP3** → 2LiI + **P3** + O_2_	+1.48	+0.28	–0.19
*id*	**S2P2** → **P3** + O_2_	+0.31	–0.01	–0.17

**Table 2:** Bromine RM Oxidation Reactions: Computed Δ_r_*G* (B2PLYP Triple-ζ Basis Set, in eV) in Different Solvents

no.	reaction	Δ_r_*G*° (gas-phase)	Δ_r_*G*° (ether)	Δ_r_*G*° (DMSO)
Peroxide Oxidation (1e^–^)				
*b1*	Br_2_ + **P4** → LiBr_2_ + **SP3**	+0.10	–0.19	–0.31
*b2*	Br_2_ + **SP3** → LiBr_2_ + **S2P2**	+0.64	+0.42	+0.33
*b3*	Br_2_ + **P4** → 2LiBr + **S2P2**	+0.78	–0.23	–0.55
*b4*	LiBr_2_ + **P4** → 2LiBr + **SP3**	+0.14	–0.65	–0.88
*b5*	LiBr_2_ + **SP3** → 2LiBr + **S2P2**	+0.68	–0.03	–0.24
*b6*	LiBr_3_ + **P4** → 3LiBr + **S2P2**	+1.10	–0.11	–0.45
Superoxide Oxidation (1e^**–**^)				
*b7*	Br_2_ + **SP3** → LiBr_2_ + **P3** + O_2_	+0.95	+0.41	+0.17
*b8*	LiBr_2_ + **SP3** → 2LiBr + **P3** + O_2_	+0.99	–0.05	–0.40
*bd*	**S2P2** → **P3** + O_2_	+0.31	–0.01	–0.17

First of all, all redox reactions in the gas phase involving either RM are endoergic (10–30 kcal/mol for iodine and 3–20 kcal/mol for bromine). For iodine in particular, all reactions are energetically penalized with respect to the simple superoxide disproportionation (reaction *id*). For bromine, instead, reaction b1, which initiates the oxidation, is the least thermodynamically penalized in the gas phase.

Solvation plays a crucial role in determining the thermodynamics of the reactions involved in the discharge process. This happens because the solvent stabilization of ionic compounds such as LiI_2_ with respect to I_2_ (e.g., reactions *i1* and *i2*) energetically favors the former. In addition, solvation promotes all reactions leading to more than one ionic molecule (e.g., reaction *i4*).

For iodine, a low-dielectric-constant solvent such as ether reduces the positive Δ_r_*G*° of the gas phase, but except for reaction *i4*, the free energy remains positive. This reduction is further enhanced by a solvent with a large dielectric constant and high polarity, such as DMSO.

For bromine, the presence of a solvent essentially makes its entire chemistry exoergic (except for reactions *b2* and *b7*).

Overall, the comparison of reactions *i1*–*i8* (Table 1) and *b1*–*b8* (Table 2) suggests that bromine-mediated reactions are either less endoergic or more exoergic compared to the corresponding steps mediated by iodine. In other words, moving from I to Br significantly reduces the Δ_r_*G°* of the reactions by ∼0.5 eV (that is, ∼10 kcal/mol), except for reaction *i3*, which is reduced by ∼0.9 eV. Accordingly, it is well-known that Br_2_ is a more powerful oxidant than I_2_.

It is important to underline that for either iodine or bromine in both solvents, the partially reduced X_2_^–^ species are seen to be very reactive oxidants (reactions *i4* and *b4*), comparable to or even better than X_2_ (reactions *i1*–*i3* and *b1*–*b3*). This means that when and if the X_2_^–^ species is formed, it should exist only as an unstable intermediate because it is readily reduced to the X^–^ halide.

The oxidative powers of I_2_ and I_3_^–^ have been previously reported to be critically dependent on the electrolyte composition,^38,39^ raising uncertainty on which one is the active oxidant form of the iodine RM. Regardless of the I_2_ ⇄ I_3_^–^ equilibrium, which is dependent on the chemical potentials of the two species at equilibrium, our calculated Δ_r_*G°* show that the strongest oxidant between I_2_ and I_3_^–^ is dictated by the solvent, as motivated by the differential solvation of the neutral/ionic species.^40^ In the ether solvent, with low polarity, reaction *i1* has a positive and low Δ_r_*G°* (0.3 eV) compared to reactions *i3* and *i6*, and I_2_ is therefore expected to be the stronger oxidant. This is reversed in DMSO, where reaction *i6* is more exoergic (−0.02 eV) than reactions *i1* and *i3*, and I_3_^–^ is consequently favored to initiate the peroxide oxidation.

In ethers with small dielectric constants, once I_2_ has initiated the oxidation (reaction *i1*), the thermodynamically favored process appears to be the oxidation of peroxide **P4** to **SP3** by I_2_^–^ (reaction *i4*). The fate of **SP3** is then determined by reactions *i5* and *i8*, which have a positive but small Δ_r_*G°* of about 0.3 eV (7 kcal/mol): the former produces an additional superoxide, yielding **S2P2**, which in turn disproportionates to yield oxygen (reaction *id*); the latter involves a superoxide oxidation to give **P3** + O_2_. In either case these last steps are those involving the possible release of singlet oxygen. Still, in the case of iodine, a solvent with a large dielectric constant such as DMSO opens additional reactive channels and makes predicting the favored thermodynamic path of the entire process more difficult. The reaction is very likely initiated by I_3_^–^ through reaction *i6* that converts **P4** into **S2P2**. However, it seems likely that I_2_^–^ can still play a major role (through reaction *i4*) in transforming **P4** into **SP3**, which then evolves, as before, toward the oxygen release through either reaction *i5* or *i8*, which has now become exoergic due to the stabilizing effect of the solvent.

In the case of bromine (Table 2), diatomic Br_2_ remains the strongest oxidation initiator, with reaction *b3* being more exoergic than reaction *b6* in ether (−0.23 vs −0.11 eV) and in DMSO (−0.55 vs −0.45 eV). Again, also for bromine, the most effective species in the oxidation of peroxide is transient Br_2_^–^. The pathways identified above for iodine are still effective also for bromine, but due to the exothermicity of many other reaction processes, it turns out that the chemistry of bromine is much less reversible than that of iodine.

### 3.2 Overall Oxidation Process

Based on the free energies reported in Tables 1 and 2, different oxidation paths can be traced from the reactant **P4** to the products **P3** + O_2_. Instead of presenting all of the possible combinations, we limit our discussion to those mechanisms that present a favorable free energy balance and appear to drive the overall redox process.

Although, as mentioned before, the bromine chemistry turns out to be much less reversible compared to that of iodine due to the concurrent presence of several exoergic processes, we also see that the I and Br chemistries are determined by the same set of pathways. Hence, for the sake of conciseness, we describe them using iodine.

**Figure 1:**
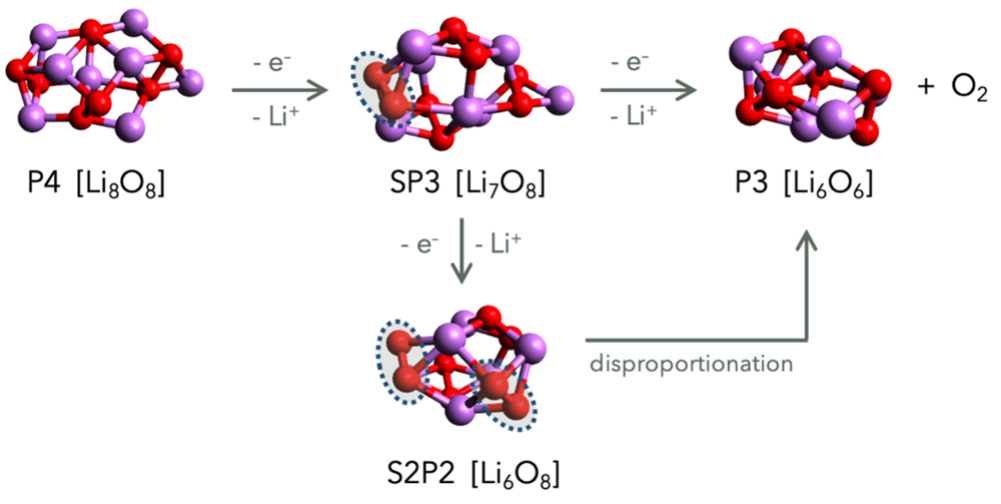
Schematic representation of the different oxidation paths leading from the **P4** reactant cluster to the **P3** + O_2_ products. The top sequence from left to right is the ET–ET mechanism, and the down branch illustrates the ET–ET–disp and DET–disp ones (see the main text for details). The dashed circles identify the superoxide units in the mixed clusters.

**Figure 2:**
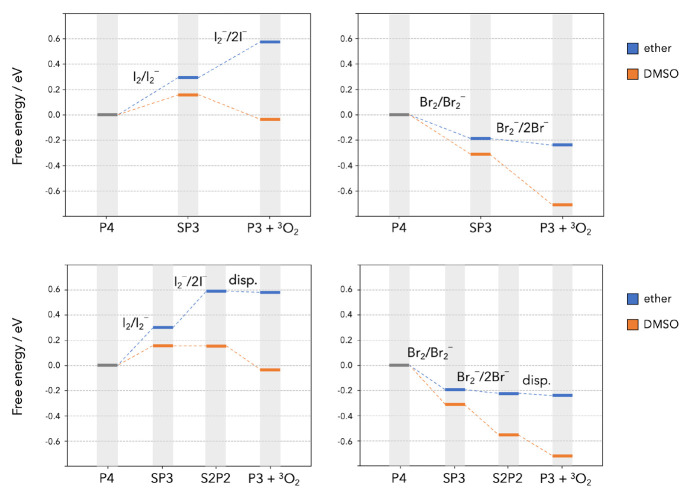
Free energies of reaction along the oxidation path of the **P4** cluster following the ET–ET mechanism (top panels) and the ET–ET–disp mechanism (bottom panels).

**Figure 3:**
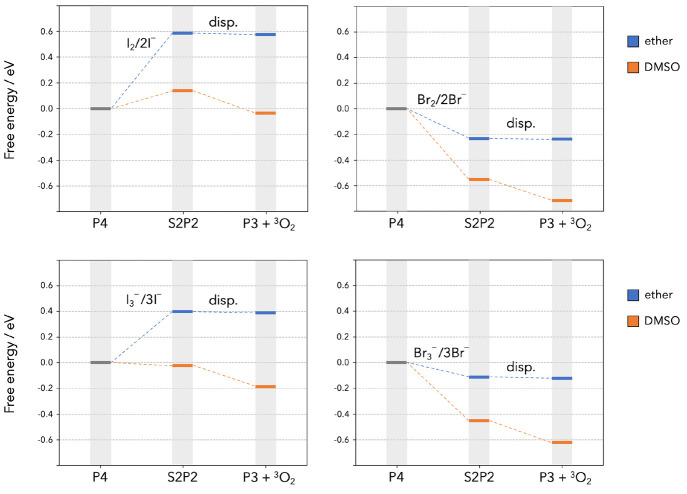
Free energies of reaction along the oxidation path of the P4 cluster following the DET–disp mechanism initiated by X_2_ (upper panels) or X_3_^–^ (lower panels).

The branched reaction sequence is shown schematically in Figure 1: a **P4** cluster is initially oxidized to form **SP3** (one-electron abstraction), which can happen through reaction *i1*. Since the resulting I_2_^–^ intermediate is a more energic oxidant than I_2_, it can either react again with the partially oxidized substrate **SP3** or with **P4** through reaction *i4*, yielding another **SP3**. The difference in the Δ*G* values of reactions *i2* and *i5* rules out the possibility that I_2_^–^ can be replaced by I_2_ to carry on the oxidation. Therefore, the process must evolve through a second one-electron oxidation of **SP3** by I_2_^–^ that produces **P3** and releases O_2_. We summarize this first sequence (ET–ET) made by two distinct subsequent electron transfers as
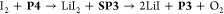
10Alternatively, I_2_^–^, in the second oxidation, can attack another superoxide to form **S2P2**, which can disproportionate, leading to the final products as in the following sequence (ET–ET–disp):
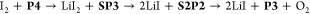
11As mentioned above, O_2_ is generally reported not to form directly by two-electron abstraction from Li_2_O_2_, a fact which may be ascribed to large kinetic barriers involved in a multielectron transfer. Therefore, a two-electron reduction of I_2_ or I_3_^–^ to form two or three I^–^ anions (eqs 7 and 9, respectively) can only take place through a pathway that sees the peroxide cluster **P4** reduced to the **S2P2** product through reaction *i3*. This reaction can then lead to O_2_ release by disproportionation of the two superoxides (DET–disp). Two different processes are possible, initiated by either I_2_ or I_3_^–^:
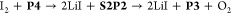
12
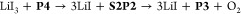
13The free energy diagrams of the ET–ET and ET–disp mechanisms are shown in Figure 2, and those of the two DET–Disp mechanisms are shown in Figure 3. In each case, the plots show the free energies calculated in both solvent models and report iodine reactions on the left and bromine reactions on the right. The final products of all eight reaction paths in Figures 2 and 3 are O_2_ + **P3**. The parasitic release of singlet oxygen can be assumed to take place at the final stage of the reactions when O_2_ is produced. Our calculations show how its production can be heavily impacted by different choices of the RM and solvent. Both the ET–ET and DET–disp overall sequences for iodine are highly endoergic in ether, and one can safely assume that ^1^O_2_ release is extremely unlikely along those paths. The same sequences become more energetically viable in DMSO, and the DET–disp sequence initiated by I_3_^–^ (bottom left panel in Figure 3) is exoergic by ∼0.2 eV. As a difference with iodine, all the hypothesized sequences (ET–ET and DET–disp) are highly exoergic for bromine regardless of the solvent dielectric properties.

In the ET–ET mechanism (Figure 2, top panels), the second oxidation step that converts X_2_^–^ into 2X^–^ is responsible for the production of O_2_. Its Δ*G* is critically influenced by the nature/polarity of the solvent, and in a much more drastic way than for the first step: when moving from diethyl ether to DMSO, the Δ_r_*G°* of the second step is seen to drop from +0.28 eV to −0.19 eV for iodine and from −0.05 eV to −0.40 eV for bromine. We ascribe this to the different solvation properties of the ionic couple LiX, which is expected to be favored by a more polar solvent. A high-polarity solvent with bromide RM will therefore provide an exoergic path toward O_2_ formation, thus making the energy barrier for singlet oxygen formation to be significantly lower than the ^3^O_2_ → ^1^O_2_ energy difference of 0.97 eV. In a less polar solvent, this effect can be greatly reduced.

When the O_2_ release step is associated with superoxide disproportionation of the partially oxidized discharge product (the **S2P2** cluster), as in the ET–ET–disp mechanism (Figure 2, bottom panels) and the DET–disp ones (Figure 3), the disproportionation step is less sensitive to the solvent. For example, the Δ*G* for this process is calculated to be −0.01 eV in diethyl ether and −0.17 eV in DMSO for iodine. Hence, the disproportionation steps should be less sensitive to solvent changes when it comes to ^1^O_2_ formation. Nevertheless, the solvent polarity heavily affects the entire thermodynamic profiles of the ET–ET–disp and DET–disp mechanisms, making them far more exoergic in highly polar DMSO. This time the major difference arises during the formation of the **S2P2** intermediate, whose solvation is strongly polarity-dependent, prior to the disproportionation step.

Spin conservation also plays a role in the above mechanisms. In each of them, the total spin multiplicity of the starting reactants is that of a singlet. Looking at the spin multiplicities of the product species, a spin transition is expected to take place to release molecular oxygen in its electronic triplet ground state. In order to assess the relevance of spin–orbit coupling (SOC) effects, calculations including the relativistic SOC effects were performed for a simplified process where a halogen diatomic X_2_ reacts with a single Li_2_O_2_ molecule, following the stoichiometry of eq 4. These model calculations show that SOC due to the heavy nuclei of the halogen atoms can exert a strong impact on spin conservation during the electron-transfer process. In Figure S2 a strong mixing of singlet and triplet states takes place when one electron is transferred from Li_2_O_2_ to I_2_, with a splitting between the resulting SO-coupled states arising on the order of ∼0.1 eV. The same mixing is shown to be much weaker in the case of Br_2_, where the splitting is roughly a third (∼0.03 eV). Hence, heavy iodine atoms are predicted to more easily promote a change in spin multiplicity during the ET process, supporting the hypothesis that the heavy-atom effect could be crucial in explaining the mechanism of ^1^O_2_ suppression in halogen RMs.^41^ The heavier nucleus of the iodine atom can more easily promote a spin transition from singlet to triplet compared to bromine, due to the stronger spin–orbit coupling. Iodine RMs are consequently predicted to be more effective at suppressing ^1^O_2_ release.

## 4 Conclusions

In this work, we tackled the investigation of the complex reactivity of lithium superoxides and peroxides with typically used redox mediators such as iodine and bromine used in LOBs. By using a simplified cluster model, we determined the free energy changes of several elementary reactions that pave the complex and entangled network of processes to transform lithium peroxide into an oxygen molecule mediated by halogen (I_2_ or Br_2_) redox couples. Our analysis has been performed under vacuum and in two model solvents with the aim of understanding the impact of solvation on the reaction mechanisms.

The main outcome demonstrates that both the elementary reactive steps and the overall processes are strongly dependent on the halide nature and on solvation, which is extremely relevant to the dielectric properties of solvents in the modulation of the reaction mechanism.

In the case of iodine, the overall reaction is initiated by I_2_ or I_3_^–^, but several subsequent reactions have been attributed to the transient species I_2_^–^ that is the main agent in transforming (Li_2_O_2_)_4_ into (Li_2_O_2_)_3_ with direct oxygen release (ET–ET process). The same ion can also transform (Li_2_O_2_)_4_ into (LiO_2_)_2_(Li_2_O_2_)_2_, which is able to undergo superoxide disproportionation, releasing oxygen (DET–disp process). This second path appears to be competitive with the first one, at least in energy. In the case of iodine, both processes in ether (low polarity and low dielectric constant) have an overall positive Δ_r_*G°* balance. They are not spontaneous, and the most advantageous of them (DET–disp initiated by I_3_^–^) requires ∼0.4 eV. Also, for iodine, a solvent with a high dielectric constant and a high polarity (like DMSO) makes both processes above only slightly thermodynamically favored (Δ_r_*G°* ≈ −0.02 eV), but nevertheless spontaneous, thus perhaps pointing to a more efficient uncontrolled chemistry taking place in the electrolyte also without any external driving force (such as an applied potential).

Bromine is revealed to be a more effective oxidant toward peroxides, and both process (ET–ET and DET–disp) turn out to be exoergic (spontaneous) both in ether and in DMSO. The bromine overall reactive path is probably initiated by Br_2_ and proceeds through the same reactions as seen for iodine, efficiently mediated by the Br_2_^–^ transient species, which can either directly provide oxygen release or promote an additional superoxide formation, hence providing a route toward Li_2_O_2_ disproportionation and, again, oxygen release. In the case of bromine, in DMSO these disproportionation pathways can release in excess of 0.6 eV of free energy, thereby providing a large portion of the energy needed to promote the release of singlet oxygen (∼1 eV) and a substantial parasitic chemistry without any applied potential. Preliminary experiments confirm this trend: a complete experimental study focusing on the impact of the solvation of singlet oxygen release and redox mediation will be published separately.

Furthermore, the probability of crossing to the triplet ground state potential energy surface (that yields triplet oxygen) is a relevant aspect in the reactivity of both iodine- and bromine-mediated reactive chemistry. For iodine, the trigger is spin–orbit coupling, which turns out to be large. On the contrary, this mechanism is less effective in bromine. Hence, despite the extremely favorable thermodynamics, the lessened efficiency of the intersystem crossing might make the kinetics of the bromine-mediated exoergic processes toward triplet O_2_ slower than that with iodine, thereby making them less efficient than how much one could simply assume by the data presented above.

Overall, it appears from our calculations that a solvent with a low polarity and a low dielectric constant seems to keep the parasitic chemistry at bay, while more solvation-effective solvents may promote singlet oxygen release and spontaneous parasitic chemistry in the electrolyte system when using redox mediators such as halogen/halide redox couples.

## Supporting Information Available

SOC calculations, structure and thermochemistry of Li–O clusters, and methods comparison (PDF)
