# Supplementary material for: A Computational Study on Halogen/Halide Redox Mediators and Their Role in 1O2 Release in Aprotic Li–O2 Batteries
Source: J Phys Chem A. 2023 Oct 27;127(44):9229–35. doi: 10.1021/acs.jpca.3c05246 (PMC10641837; doi:10.1021/acs.jpca.3c05246)
Supplement: Supplementary file 1 — jp3c05246_si_001.pdf [file jp3c05246_si_001.pdf]

Supplementary Information

A Computational Study on the Halogen/halide Redox Mediators and their Role in the $^1O_2$ Release in Aprotic Li-O_2 Batteries

*Adriano Pierini[a], Angelica Petrongari[a], Vanessa Piacentini[a], Sergio Brutti[a], Enrico Bodo[a]**

[a] Chemistry Department, University of Rome "La Sapienza", Rome, Italy

*Corresponding Author email: enrico.bodo@uniroma1.it

S1. SOC calculations

The calculations were performed using DFT with the scalar-relativistic DKH2 Hamiltonian, together with the all-electron basis set DKH-def2-TZVP (SARC-DKH-def2-TZVPP for iodine). Single-point TDDFT energies were calculated for the first 3 singlet and 3 triplet excited states, using the ωB97X-D range-separated hybrid functional [1] along suitable scans of the potential energy surface. This "range separated" functional was chosen for its remarkable improvements over more common hybrid functionals when dealing with charge-transfer excited states [2,3]. Spin-orbit coupling between states at different multiplicities has been evaluated by quasi-degenerate perturbation theory [4], as implemented in the ORCA package.

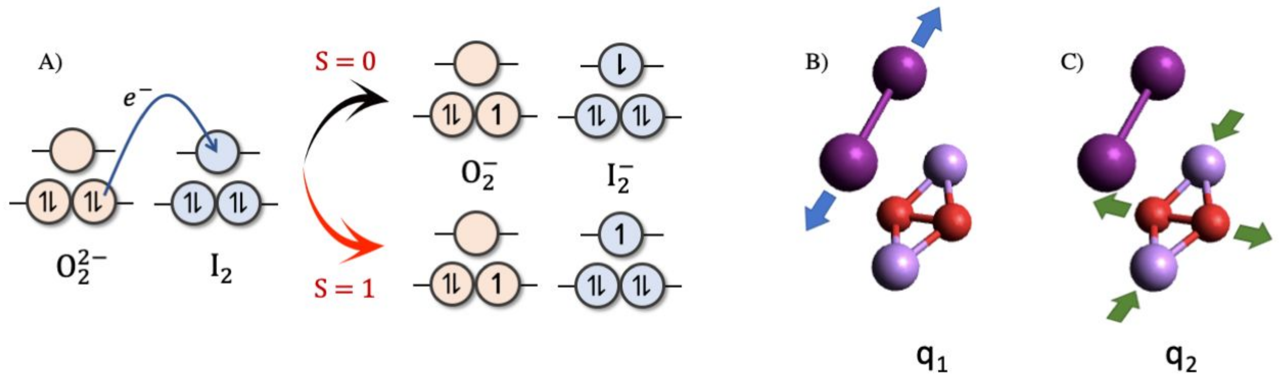

Figure S1. A) The main excitation character of roots S1 and T1 is represented by a oxygen-to-halogen charge transfer, thus leading to the one-electron oxidation product of Li_2O_2. B) The q_1 vibrational normal mode is associated to the X-X bond stretching; a positive step along q_1 hence stabilizes the X_2^- electronic state over X_2. C) The q_2 vibrational normal mode is associated with the O-O bond stretching and the bending of the O-Li-O angles; a negative step along q_2 hence destabilizes the peroxide electronic state favoring the superoxide state.

Harmonic frequencies have been calculated in the minimum-energy, optimal geometry of the complex, and the vibrational normal modes have been analyzed. Based on our previous insights on CASSCF-computed potential energy surfaces for the disproportionation of the superoxide anion [5,6], we focused on the two normal modes that characterize the electron-transfer reaction coordinate: q_1 (eigenvector n. 12 of the Hessian matrix), corresponding to the stretching of the X-X bond (purple spheres in Figure S1), and q_2 (eigenvector n. 15 of the Hessian matrix), corresponding to the stretching of the O-O bond coupled with a symmetrical bending of the two O-Li-O angles in the superoxide (Figure S1, right panel).

The minimum-energy structure was then projected along both q_1 and q_2, where the magnitude of the displacements taken in both directions has been expressed as a fractional multiple of the eigenvector. This is the scale unit reported in the *x*-axis of figures S2 and S3. A grid of geometries along the q_1 and q_2 coordinates has been generated, for each of which the TDDFT excited states were computed yielding the potential energy surfaces which are reported in Figure S2 and S3 as cuts along one of the two normal modes. In the top two panels (a and b) of the TDDFT, spin-pure electronic states are reported for a selected, indicative value of the other normal mode. A crossing is clearly visible between the ground-state singlet S0 (blue curve), corresponding to the reactant configuration and the first excited triplet T1 (violet curve) that is the oxidation product after a first one-electron abstraction where, in fact, the most important excitation corresponds to an oxygen-to-halogen electron transfer. The bottom panels (c and d) show a magnification of the crossing regions, now with the inclusion of the SO-coupled states which arise by their mixing (these states are indicated as SOC1 and SOC2).

More specifically, they are the result of the Ms = 0 only spin component of the singlet state S0 interacting with the Ms = ±1 components of the triplet state T1.

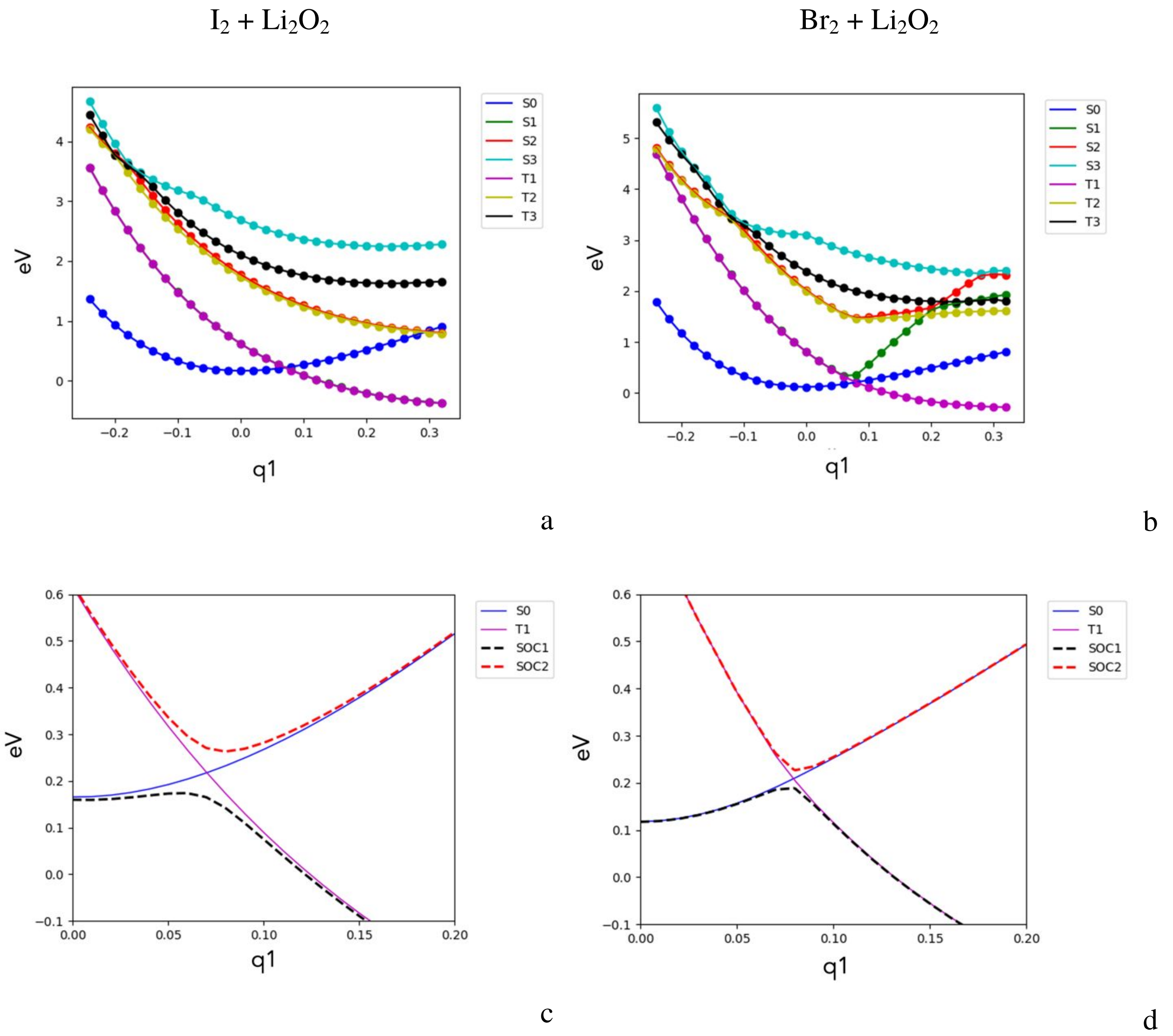

Figure S2. Potential energy surfaces along the q_1 coordinate, cut at q_2=-0.10. Panels a,b show the computed TDDFT spin-pure states. Panels c and d show a magnification of the crossing region between states S0 and T1, plotted together with the SOC states.

$I_2 + Li_2O_2$ $Br_2 + Li_2O_2$

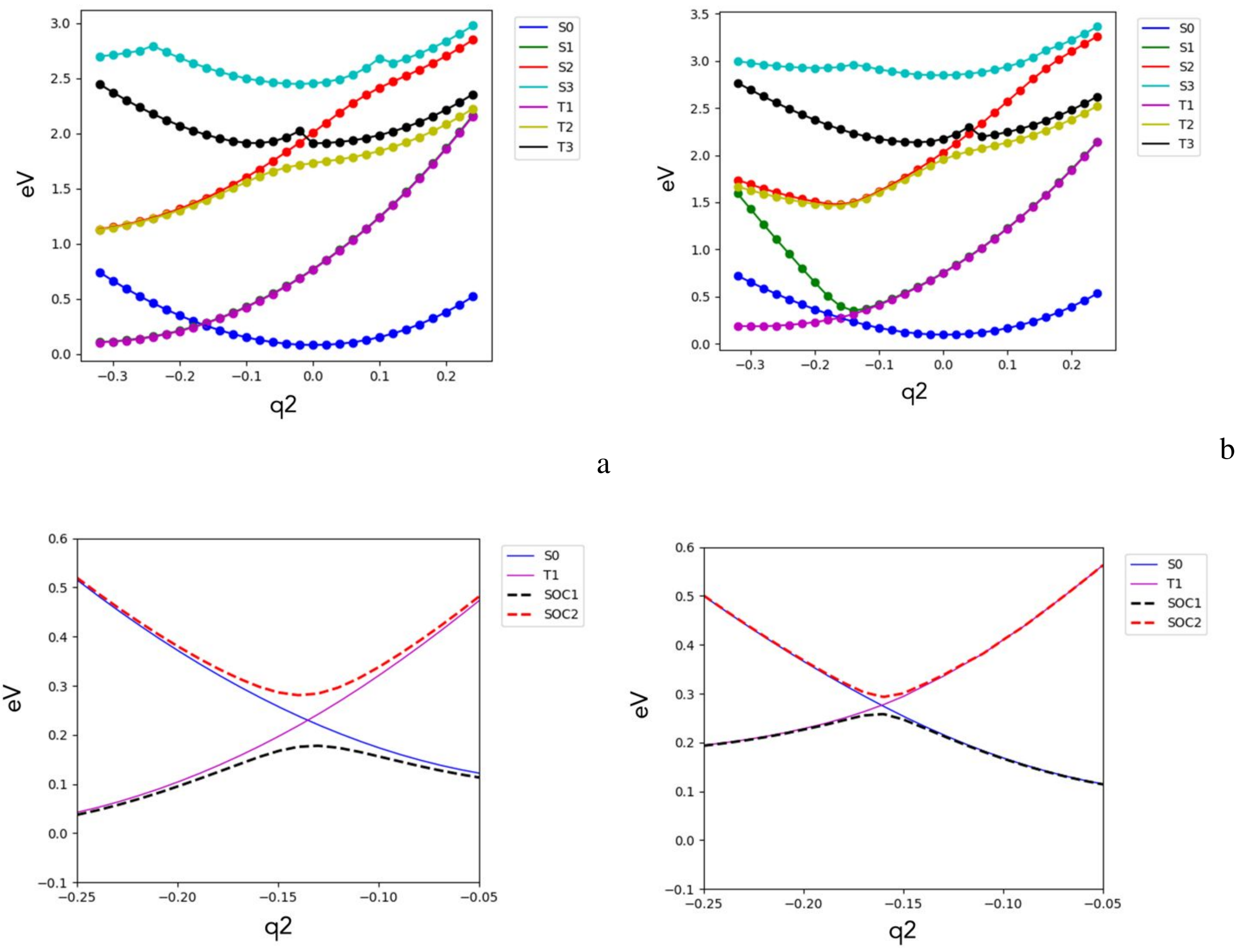

a b c d

Figure S3. Potential energy surfaces along the q_2 coordinate, cut at q_1=+0.04. Panels a,b show the computed TDDFT spin-pure states. Panels c and d show a magnification of the crossing region between states S0 and T1 energy splitting is clearly recognizable due to avoided crossing.

S2. Structure and Thermochemistry of Li-O clusters

Cluster	Energy function [Eh]	XYZ coordinates (in vacuo) [Å]
**P4**<br>[Li_8O_8]<br>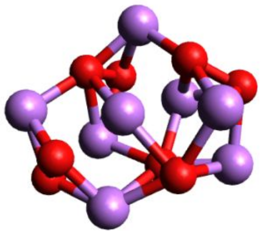	In vacuo<br>E0 = -662.59270113<br>H = -662.53342733<br>G = -662.58844145<br>SMD diethyl ether<br>E0 = -662.62717198<br>H = -662.56875035<br>G = -662.62459781<br>SMD dmso<br>E0 = -662.63742321<br>H = -662.57927946<br>G = -662.63544999	X Y Z<br>Li 0.07274 0.81473 -1.08034<br>O 1.01356 2.37184 -1.20252<br>O 1.84693 1.08159 -0.98753<br>Li 1.68126 0.81552 0.92380<br>Li 2.92425 2.74993 3.09129<br>O 1.98119 2.75140 1.55469<br>O 3.29019 1.93720 1.48445<br>Li 2.67195 2.55769 -0.30097<br>Li -0.34984 0.00994 2.46101<br>O 2.04917 1.25543 3.98952<br>O 1.47667 0.24440 2.97713<br>Li 3.22459 0.46556 2.81655<br>Li 0.17700 2.58635 0.47946<br>O -0.82477 1.69570 1.81287<br>O -0.29258 0.68168 0.79139<br>Li 0.72475 1.97810 2.87536
**SP3**<br>[Li_7O_8]<br>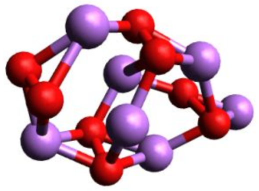	In vacuo<br>E0 = -654.97112500<br>H = -654.91638301<br>G = -654.97649468<br>SMD diethyl ether<br>E0 = -655.00330031<br>H = -654.94934408<br>G = -655.01027533<br>SMD dmso<br>E0 = -655.01337257<br>H = -654.95964367<br>G = -655.02089913	X Y Z<br>Li 0.39080 0.38311 3.48764<br>O 2.06191 0.99184 3.10455<br>O 1.60646 -0.14708 2.17405<br>Li 3.19979 0.58697 1.65099<br>Li 0.36555 0.38233 0.93337<br>O 0.12653 1.90510 -0.06803<br>O 0.59342 3.00021 0.90784<br>Li 1.89150 2.40568 -0.30274<br>Li 1.94339 2.57307 2.11601<br>O 3.46679 1.35590 -0.07552<br>O 3.55026 2.50957 0.96980<br>Li 4.90365 2.35792 -0.10895<br>Li -1.07380 2.42095 1.28978<br>O -1.07477 1.66364 3.13388<br>O -1.24841 0.63988 2.25527
**S2P2**<br>[Li_6O_8]<br>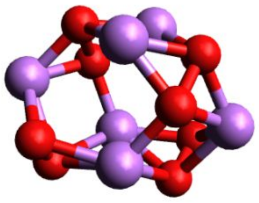	In vacuo<br>E0 = -647.33753802<br>H = -647.28660713<br>G = -647.34475909<br>SMD diethyl ether<br>E0 = -647.36431155<br>H = -647.31405967<br>G = -647.37330962<br>SMD dmso<br>E0 = -647.37354091<br>H = -647.32338876	X Y Z<br>Li 0.17700 0.48796 0.62282<br>O 1.25353 0.83007 -1.00601<br>O 0.15178 1.62571 -0.93183<br>Li 1.77053 2.79042 -0.84678<br>Li 2.72569 0.63706 0.54428<br>O 1.68912 -0.74781 1.51017<br>O 2.74368 -0.28154 2.23250<br>Li 1.05729 0.17653 3.19854<br>Li 2.46955 2.72962 2.34699<br>O 1.77326 3.53162 0.80348<br>O 2.87004 2.48581 0.60545

	G = -647.38284265	Li 0.24673 3.00600 1.77662<br>O -0.07977 1.30332 2.27349<br>O 0.95143 1.98120 3.17594
**P3**<br>[Li_6O_6] 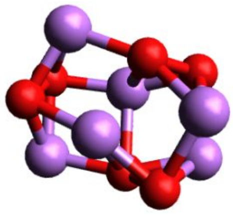	In vacuo<br>E0 = -496.90909208<br>H = -496.86490078<br>G = -496.91123327<br>SMD diethyl ether<br>E0 = -496.94095956<br>H = -496.89761120<br>G = -496.94479546<br>SMD dmso<br>E0 = -496.95055177<br>H = -496.90746275<br>G = -496.95494264	X Y Z<br>Li 1.73784 2.29435 0.74359<br>O 0.41567 1.63184 -0.37065<br>O 1.34856 0.51803 0.14893<br>Li -0.37560 0.23837 0.48196<br>Li -0.60022 2.46076 1.17254<br>O -0.43376 1.06408 2.32075<br>O 0.62102 2.18359 2.43321<br>Li 1.29615 0.41762 2.17046<br>Li 3.25282 0.53344 0.57426<br>O 3.39584 2.02583 1.62123<br>O 3.15642 0.70812 2.39121<br>Li 2.43212 2.19310 3.16883

Table S1. Calculated thermochemical functions (in Hartree) and optimized geometries of the four Li-O clusters: total electronic energy (E_0), enthalpy (H) and Gibbs free energy (G) calculated at DFT/B2PLYP level in the three different environments (gas phase and the two implicit solvents). Optimized geometry in the gas-phase, in the XYZ format (units are in Å).

S3. Methods comparison

The potential (electronic) energy of selected reactions were computed with different methods. All calculations employed the DKH scalar-relativistic Hamiltonian with a suitable all-electron basis set: DKH-def2-TZVPP (on Li and O) and SARC-def2-TZVPP (on I) for the DFT functionals ωB97X-D3 and B2PLYP, while cc-pVTZ-DK for coupled cluster. CCSD(T) single-point energy were calculated on top of MP2-optimized geometries (same basis set).

Table S2. Formation energies (in Hartree units) for the peroxide/superoxide clusters, calculated as E = E(cluster) - Σ E(monomers).

Cluster	ωB97X-D3	B2PLYP + D3	CCSD(T)
P4	-0.275306	-0.292827	-0.307874
SP3	-0.257161	-0.270662	-0.278408
S2P2	-0.225679	-0.236486	-0.242916
P3	-0.172717	-0.184186	-0.194771

Table S3. Reaction electronic energy (in Hartree units) for selected oxidation reactions.

Reaction	ωB97X-D3	B2PLYP + D3	CCSD(T)
I_2 + P4 → LiI_2 + SP3	0.022738	0.032396	0.042515
LiI_2 + SP3 → 2LiI + P3 + O_2	0.070639	0.082235	0.074959
LiI_2 + SP3 → 2LiI + S2P2	0.043083	0.059421	0.058851
LiI_3 + P4 → 3LiI + S2P2	0.064985	0.103476	0.102708
S2P2 → P3 + O_2	0.027556	0.022814	0.016107
